# Alcohol Consumption and Cervical Carcinogenesis: Time to Draw Conclusions

**DOI:** 10.3390/cells14201639

**Published:** 2025-10-21

**Authors:** Vivek K. Kashyap, Divya B. Kenchappa, Ajay K. Singh, Bhupesh Singh, Murali M. Yallapu, Everardo Cobos, Subhash C. Chauhan

**Affiliations:** 1 Division of Cancer Immunology and Microbiology, Medicine and Oncology Integrated Service Unit, School of Medicine, University of Texas Rio Grande Valley, McAllen, TX 78504, USA; murali.yallapu@utrgv.edu; 2 South Texas Center of Excellence in Cancer Research (ST-CECR), McAllen, TX 78504, USA; 3 Department of Pharmaceutical Sciences, University of Tennessee Health Science Center, Memphis, TN 38163, USA; divya.hosamane@gmail.com; 4 Department of Pediatric-Pulmonology, University of Tennessee Health Science Center, Le Bonheur Children’s Hospital, Memphis, TN 38103, USA; ajay.25.singh@gmail.com; 5 School of Applied Sciences, OM Sterling Global University, Hisar 125001, Haryana, India; bhupeshsingh20011996@gmail.com; 6 Department of Medicine, School of Medicine, University of Texas Rio Grande Valley, McAllen, TX 78504, USA; everardo.cobos@utrgv.edu

**Keywords:** cervical cancer, alcohol consumption, immune response, smoking, HIV/HPV co-infection, risk factors

## Abstract

Cervical cancer is the fourth most common cancer among women worldwide and remains a significant cause of cancer-related mortality. Alcohol consumption is linked to an increased risk of several cancers and is a controversial risk factor for developing cervical cancer. This review updates existing information on the correlation between alcohol consumption and the risk of developing cervical cancer. Several comprehensive studies from different geographical regions have shown that moderate and heavy drinking is positively correlated with the development of cervical cancer. There is a synergistic relationship between human papillomavirus (HPV) viral load and alcohol use among drinkers with a high HPV viral load. Excessive alcohol consumption and exposure to second-hand smoke may elevate the risk of persistent HPV infection. Furthermore, high-risk behaviors associated with Human immunodeficiency virus (HIV)/HPV co-infection are more common among binge drinkers. However, several observations failed to establish a relationship between these factors. Despite some inconsistency in the literature, evidence suggests a modest association between alcohol consumption and increased risk of persistent HPV infection, causing cervical cancer.

## 1. Introduction

Cervical cancer is the fourth most common cancer in women worldwide, accounting for 660,000 new cases and 94% of 350,000 deaths in low- and middle-income countries in 2022 [1,2]. Low- and middle-income countries have the highest rates of cervical cancer incidence and mortality due to inequities in access to national human papillomavirus (HPV) vaccination, screening, and treatment services, as well as social and economic determinants [1,3]. The main preventable risk factors for cervical cancer include HPV infection, multiple sex partners, smoking, immunocompromised status (e.g., AIDS), chlamydia infection, long-term use of oral contraceptives, having numerous full-term pregnancies, young age at first full-term pregnancy, and low economic status [4].

Alcohol consumption is the third most significant modifiable cancer risk factor after tobacco use and excess body weight [5]. It is a recognized cause of at least seven types of cancer [6]. Alcohol is classified as a Group 1 carcinogen by the International Agency for Research on Cancer (IARC) of the World Health Organization (WHO) [7]. Addressing high-risk alcohol consumption is a crucial strategy for reducing the burden of cancer. Alcohol is a modifiable risk factor for several cancers, and reducing or eliminating alcohol use can significantly lower the risk of developing these diseases [8].

Alcohol has several direct and indirect effects that underpin carcinogenesis, affecting genetic, epigenetic, biochemical, and immunological defects and causing chronic inflammation and carcinogenesis [9]. Alcohol metabolites are either oxidative or nonoxidative, yielding acetaldehyde and other metabolites [10]. Alcohol influences carcinogenesis via its metabolites through several mechanisms [9,11]. These mechanisms lead to genetic, epigenetic, biochemical, and immunological defects, resulting in chronic inflammation and carcinogenesis [9,11]. Research on the association between alcohol use and cancer risk in the cervix, uterus, vulva, and vagina has shown conflicting or inconclusive findings [12,13]. However, the potential role of alcohol consumption in the etiology of cervical cancer has not been extensively studied. This comprehensive review article highlights the most current epidemiological evidence studies examining alcohol consumption patterns and cervical cancer risk, molecular mechanisms underlying alcohol-induced carcinogenesis, alterations in alcohol-mediated tumor immunology responses, and synergistic effects of alcohol intake combined with smoking and HPV-HIV co-infections in cervical cancer progression and clinical development outcomes.

## 2. Alcohol and Cancer

**Figure 1:**
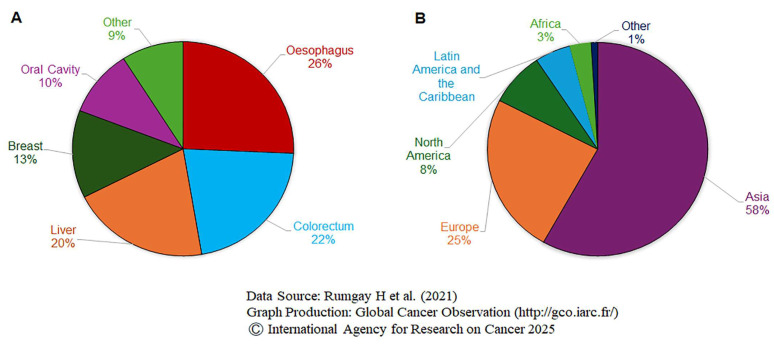
The number of new cancer cases worldwide in 2020 may be attributed to alcohol, both sexes using the data shown by cancer site (**A**) and population (**B**). The data sources are Rumgay H et al. (2021) [16], Graph production: Global Cancer Observatory (http://gco.iarc.fr/), Accessed on 19.08.2025. © International Agency for Research on Cancer 2025.

In 2012, alcohol caused 5.5% of all new cancer cases and 5.8% of all cancer-related deaths globally [14], with approximately 3.5% of cancer deaths in the US attributed to alcohol consumption [15]. The distribution of the estimated number of cancer cases in 2020 attributed to alcohol consumption by site was shown in Figure 1A, while Figure 1B presents the same distribution by population group by both sexes[16]. Between 2016–2017 and 2020–2021, annual U.S. deaths attributed to excessive alcohol use rose sharply from about 138,000 to 178,000, an increase of over 40,000 deaths (29%) [17]. The link between alcohol consumption and cancer remains complex and somewhat controversial, depending on the site of malignancy. The relationship between alcohol consumption and cancer risk is not uniform across all types of cancer, and some associations are stronger than others [18]. Alcohol is a well-established risk factor for cancers of the oral cavity, pharynx, esophagus, colorectum, liver, larynx, and female breast. However, its role in other malignancies is less clear. Some studies suggest an association with pancreatic, prostate, and melanoma cancers [19,20].

Elderly individuals, especially women, are at greater risk due to gender-related metabolic variances and age-related physiological differences [21]. Alcohol consumption has been linked to an increased risk of cancer mortality in men, with an average risk ratio of 1.53 [21]. This increase is evident in men who consume three to five alcoholic beverages per day, while women who consume multiple drinks have a risk ratio of 1.23 [22]. Even moderate alcohol consumption can raise the risk of cancer, with severe, long-term usage posing the most significant hazards [8]. Aside from oncology, alcohol consumption and abuse are serious public health issues. Population studies show that around 12% to 14% of adults currently suffer from alcohol use disorder, and 29% have experienced such a disorder in their lifetime [23]. The risk of cancer increases with higher alcohol consumption, regardless of the type of alcohol consumed [24]. Other metrics used to evaluate the effect of alcohol include excessive drinking, binge drinking, and heavy drinking. Excessive drinking, also known as binge drinking, is characterized by consuming four or more drinks in one sitting for women and five or more drinks for men. Binge drinking is the most prevalent form of excessive drinking, compared to heavy drinking, which includes eight or more drinks per week or three or more drinks per day for women. Over the past decade, severe binge drinking among adults has increased, with over four to five drinks consumed daily [25]. Moderate drinking is defined as one drink per woman and two drinks per man [14,26], but most excessive drinkers do not meet alcoholism or dependency criteria [26,27]. Compromised alcohol-induced immunity heightens vulnerability to infections, including bacterial pneumonia, septicemia, tuberculosis, and hepatitis [28]. However, limited research and epidemiological evidence demonstrate associations between alcohol intake and cervical cancer.

## 3. Human Papillomavirus and Other Risk Factors in Cervical Cancer

Cervical cancer development from transient HPV infection depends on immune status, sexually transmitted diseases, reproductive factors, contraceptives, genetics, and host factors [29]. HPV represents a significant global health challenge, with high-risk strains accounting for 10.4% of worldwide infections and reaching rates as high as 36.5% in certain developing regions. [30,31]. HPV is primarily acquired after sexual activity, often transmitted to the vaginal canal, with higher chances of transmission with multiple partners or a history of other sexually transmitted infections [32,33]. Chlamydia trachomatis infection increases cervical squamous cell carcinoma risk, with DNA detected in 40% of cases [31]. Herpes simplex virus type 2 (HSV-2) seropositivity significantly correlates with invasive cervical cancer (ICC) development [34]. Combined oral contraceptive (OC) use is strongly associated with increased cervical cancer risk [35]. Research demonstrated that obese and overweight women face twice the risk of developing cervical adenocarcinoma compared to average-weight women [36]. Smoking represents a critical cervical cancer risk factor, with nearly double the risk for smokers versus non-smokers [37,38,39]. Tobacco causes local immune suppression and DNA damage in squamous cells [40]. Persistent high-risk HPV infections drive cervical cancer development, with malignant transformation typically occurring at the cervical transformation zone, where metaplastic epithelial changes create vulnerable tissue [40]. Age is also an essential determinant of the risk of HPV infection [41]. The peak risk is observed during puberty and pregnancy. Following menopause, this risk tends to decline and may be attributed to a decrease in sexual activity, which consequently leads to lower exposure to the virus [42]. Approximately 30 HPV strains are transmitted sexually and infect genital areas, including the cervix, vagina, vulva, penis, scrotum, rectum, and anus. HPVs are categorized as high-risk or low-risk types based on their association with cervical cancer and precursor lesions [43]. HPV types 6, 11, 42, 43, 44, 54, 61, 70, 72, and 81 are low-risk types, while 16, 18, 31, 33, 35, 39, 45, 51, 52, 56, 58, 59, 66, 68, 73, and 82 are high-risk or oncogenic types [44].

**Figure 2:**
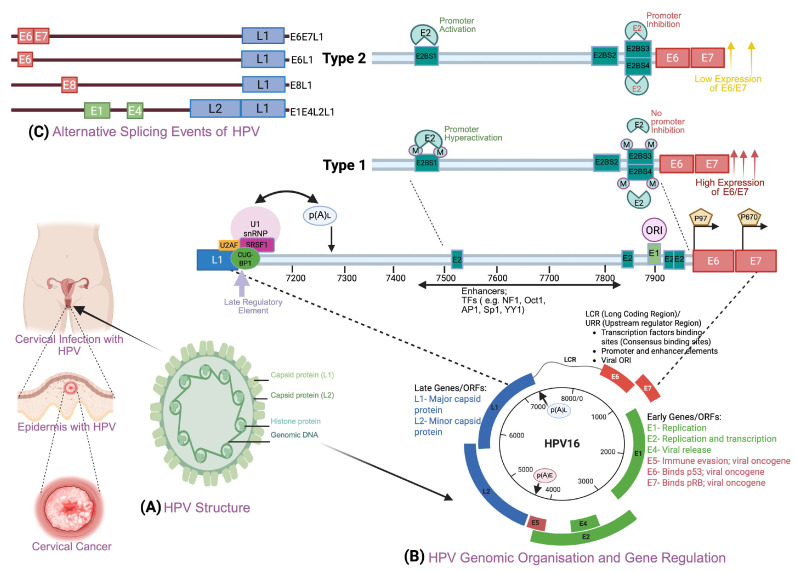
HPV structure, genomic organization, and gene regulation. (**A**) HPV Structure: HPVs are small, non-enveloped viruses made of double-stranded DNA, icosahedral in shape and measuring 7.9 kilobases. They have eight major genes, six of which are open reading frames (ORFs): E1, E2, E4, E5, E6, and E7, and two genes, L1 and L2, in the late regions. (**B**) HPV genome organization and gene regulation: red and green represent early proteins; blue represents late proteins; LCR/upstream regulatory region (URR); p(A)L, late polyadenylation signal; p(A)E, early polyadenylation signal. The early proteins are essential for viral replication, transcription, and cellular regulation, including apoptosis and immune modulation. The LCR, positioned between early and late regions, harbors four E2 binding sites where transcriptional regulator E2 binds, while replication protein E1 associates with the origin of replication (ORI) to initiate viral DNA synthesis. Many cellular transcription factors, including the E2 dimer, help regulate viral early gene expression by binding to the LCR’s regulatory sequences. Type 1) The methylation on LCR at E2BS1, towards the 3′ ends (downstream) of the L1 open reading frame, is shown in blue, and the E6 gene located upstream at the 5′ end is shown in red. The LCR has four highly conserved E2BS (E2BS1, E2BS2, E2BS3, and E2BS4) DNA sequences. E2BS2 methylation results in the E2 regulatory protein no longer inhibiting the promoter, and the binding of TFs hyperactivates the promoter p97, leading to a high level of E6/E7 oncoproteins. Type 2) In unmethylated E2BS, the E2 protein activates the p97 promoter by binding to the high-affinity E2BS1 site, leading to low-level expression of the E6/E7 early genes. (**C**) Alternate Splicing Events: HR-HPV oncogenic E6/E7 mRNA splicing is regulated by various mechanisms. A group of proteins called the spliceosome cuts the mRNA by removing the non-coding introns from a pre-mRNA and joining the exons, making the final mRNA that codes for proteins. The introns are marked by a 5′ splice site (splice donor) and a 3′ splice site (splice acceptor). This image was created with BioRender.com on 20 May 2025 (BioRender.com/ma29rh9*)*.

HPV, a member of the Papillomaviridae family, is a small, non-enveloped, icosahedral virus. The genome of the virus is a circular double-stranded DNA, containing approximately 8000 bp, organized into three regions: the long control region (LCR), the early (E) region for viral replication products, and the late (L) region for structural proteins [44,45,46] (Figure 2). The first is a “non-coding upstream regulatory region” known as the long control region (LCR) or upstream regulatory region (URR). Positioned between L1 and E6 genes, the LCR houses the p97 early promoter controlling E6/E7 transcription and DNA replication [47].

The LCR is approximately 1000 base pairs long and consists of three sub-segments: the 5′ segment, which contains transcription termination signals and a nuclear matrix binding region; the central segment, which contains transcription factors regulating viral gene expression; and the 3′ segment, which includes the early promoter and replication origin [48]. The second is called the early (E) region, which comprises 6–8 open reading frames encoding essential E1, E2, E5, E6, E7, and E1^E4 proteins that facilitate viral replication and cellular oncogenesis processes. The third is referred to as the “late region (L),” which produces critical L1 and L2 capsid proteins, designated as major and minor structural components, respectively, which mediate viral entry into susceptible host cells during the initial infection process [49]. HPV establishes productive infections by invading the mucocutaneous epithelium, typically through microabrasions, and subsequently replicates within mature epithelial cells [50]. HPV integrates its viral DNA into the host genome, disrupting cellular genes and normal cell cycle regulation mechanisms. This integration can lead to the deletion of E2 ORF and stimulate unregulated cell division, ultimately resulting in the accumulation of genetic abnormalities [51]. Furthermore, E6 and E7 oncoproteins are essential for infected cells to enter mitosis and amplify the HPV genome, partly dependent on the cell division program. While E6 and E7 oncoproteins are essential for cancer development, their expression is required for HPV replication [52]. E7 drives cell cycle progression by facilitating G1-S phase transition through interaction with tumor suppressor pRb and Cullin 2 ubiquitin ligase, resulting in pRb degradation. This process releases transcription factor E2F from pRb inhibition, promoting expression of target genes, including cyclin E, cyclin A, and p16INK4A [53]. Meanwhile, E6 interacts with tumor suppressor p53, forming a complex with E6-associated protein (E6AP) that promotes p53 degradation via the proteasome pathway [54]. This mechanism inhibits p53 functions such as apoptosis induction and DNA damage repair [53]. Additionally, both E6 and E7 activate multiple signaling pathways that facilitate viral replication and maintain the cellular environment necessary for continued HPV genome replication [55].

## 4. Molecular Mechanism of Alcohol-Mediated Carcinogenicity

**Figure 3:**
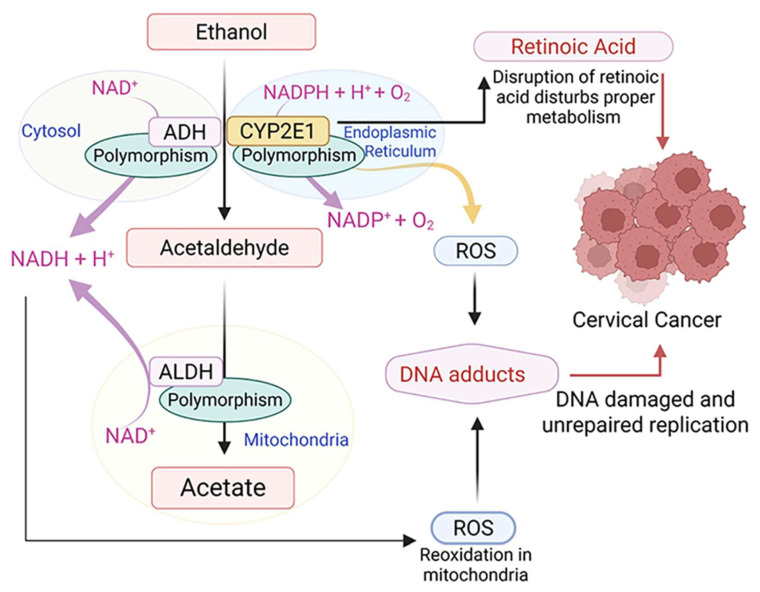
Molecular mechanism of alcohol-mediated carcinogenicity and metabolism. Cytosolic ADH converts ethanol to acetaldehyde, which is then oxidized by the mitochondrial ALDH enzyme to form acetate. The endoplasmic reticulum (ER) also oxidizes ethanol, producing ROS. The mitochondria reoxidize NADH to NAD+, which may lead to increased ROS generation. Acetaldehyde and ROS can bind to DNA, forming DNA adducts—polymorphisms in ALDH cause varying amounts of aldehyde production and oxidation. A CYP2E1 polymorphism increases the production of ROS, which are involved in carcinogen metabolism. Excessive alcohol consumption can cause changes in the CYP2E1 enzyme, disrupting the balance of retinoids and potentially contributing to cancer development. This image was created with BioRender.com on 20 May 2025 (BioRender.com/z5l6teh).

The link between alcohol use and cancer risk has been investigated through various pathways, with key mechanisms discussed in Figure 3 to explain that the carcinogenic effects of alcohol consumption might also relate to cervical cancer. Ethanol metabolism involves both oxidative and non-oxidative processes. Ethanol is metabolized oxidatively by key enzymes, including alcohol dehydrogenase (ADH), cytochrome P450 2E1 (CYP2E1), or bacterial catalase, which generate acetaldehyde as the primary metabolite [10,56,57]. However, there is little evidence on the molecular mechanism of alcohol-mediated carcinogenicity, especially in the context of cervical cancer.

### 4.1. Acetaldehyde Production

Acetaldehyde converts to acetic acid via aldehyde dehydrogenase (ALDH), CYP2E1, or a combination of aldehyde oxidase (AO) and xanthine oxidase (XO) [10,56,58]. This highly reactive metabolite forms DNA adducts, altering DNA structure and blocking synthesis/repair [59]. These genotoxic adducts create DNA-protein and interstrand cross-links, promoting carcinogenesis [59]. Acetaldehyde and ethanol modulate DNA methylation, affecting oncogene and tumor-suppressor expression [9]. Acetaldehyde inhibits DNA methyltransferase (DNMT) activity, reduces DNMT mRNA, and blocks S-adenosyl-L-methionine synthesis—both critical for methylation [60,61]. Studies of cervical cancer SiHa cells and tumor-bearing mouse models showed that ethanol altered methyl donor and DNMT expression, reduced intracellular DNA methylation levels, and promoted tumor growth through hypomethylation [62].

ADH polymorphisms correlate with cancer risk [63,64,65], and they encompass three gene loci (ADH1A, ADH1B, ADH1C) containing genetic variations that elevate cancer risk, particularly with heavy alcohol consumption. The ADH1B2 allele produces enzyme activity 40-fold higher than ADH1B1, rare in Caucasians but prevalent in Asians [9]. ADH1B and ADH1C variations increase alcoholism and cirrhosis susceptibility in Asians [66]. ADH1B*1/1 genotype carriers face higher risks for upper aerodigestive tract, oral cavity, hypopharynx, and ovarian cancers. Women carrying the ADH1C1 variant show 1.8-fold increased breast cancer risk, especially premenopausal women [67]. Several isozymes, such as CYP1A1 and CYP2E1, modulate genetic cancer risk, notably in colon, lung, and breast carcinomas [68,69,70,71]. Heavy alcohol use worsens HCC risk, especially in Asians with certain CYP2E1 genotypes [72]. Limited evidence currently exists linking enzyme polymorphism to alcohol-mediated carcinogen metabolism in HPV-related cervical cancer development. Cervical cancer patients show elevated serum ADH and ADH I levels versus controls [73].

Cervical cancer cells demonstrated significantly elevated class I ADH activity compared to healthy mucosa, with increased total ADH activity, while ALDH remained unchanged [74]. Furthermore, in other gynecological cancers, such as endometrial cancer, patients exhibit significantly elevated total ADH and class I ADH activities compared to healthy controls and patients with uterine myomas [75]. ADH I demonstrates moderate diagnostic potential with an AUC of 0.682 with 69% sensitivity and 77% specificity, indicating possible utility as a tumor biomarker [75]. However, this moderate diagnostic performance suggests that ADH I alone is insufficient as a standalone diagnostic test for endometrial cancer detection. The AUC value below 0.7 reflects limited discriminatory power, necessitating further investigation. These findings indicate that ADH I would be effectively utilized as a component within a comprehensive multi-biomarker panel rather than as an independent diagnostic tool.

### 4.2. Oxidative Stress

Excessive ethanol consumption induces oxidative stress, generating reactive oxygen species (ROS) and hydrogen peroxide (H_2_O_2_) that contribute to the development of various cancer, including cervical cancer [9,76,77]. ROS function as signaling molecules driving cancer initiation, promotion, and progression [78]. ROS accumulation upregulates monocyte chemotactic protein-1 (MCP-1) and vascular endothelial growth factor (VEGF), mediating tumor angiogenesis and metastasis. These factors significantly influence CIN development and progression [79]. Chronic ethanol consumption elevates hepatic CYP2E1 enzyme levels 10–20 fold, substantially increasing ROS production [76]. This leads to lipid peroxidation, generating toxic products including malondialdehyde and 4-hydroxynonenal (4-HNE) [80]. These compounds react with DNA bases, forming highly mutagenic exocyclic DNA adducts [81,82]. Such adducts can induce mutations in critical genes like TP53 (codon 249), encoding the p53 tumor suppressor protein, regulating apoptosis and cell growth [83]. DNA adduct levels depend on antioxidant defense systems, genetic polymorphisms (glutathione-S-transferase), DNA repair mechanisms, and apoptotic processes [84]. These adducts correlate with HPV infection in squamous cell cervical cancer, serving as molecular oncogenesis markers [85].

### 4.3. Altered Retinoid Metabolism

Retinoids play a significant role in regulating carcinogenesis by inhibiting cell growth, differentiation, and apoptosis [86]. Retinoids demonstrate significant anti-proliferative effects on HPV-infected cervical epithelial cells while modulating cellular differentiation pathways [87,88]. However, alcohol can alter retinoid metabolism by inhibiting vitamin A oxidation into retinoic acid and increasing cytochrome P450, family 2, subfamily E, polypeptide 1 (CYP2E1) activity, leading to toxic retinoid metabolites [89]. This may explain the excess lung cancer risk in smokers with β-carotene supplements. Prolonged alcohol intake is linked to decreased liver retinoids and a higher risk of head and neck cancers [90]. Changes in ADH and ALDH activity can disrupt the retinoid balance [91,92], resulting in abnormal cellular differentiation processes and potentially heightening susceptibility to HPV infection in cervical epithelial tissues. The reduced level of all-trans-retinoic acid (ATRA) in serum has been found to activate the progression of cervical lesions to invasive cancer [93]. However, ATRA plays a beneficial role by attenuating the progression of human cervical neoplasia through the enhancement of the secretion of transforming growth factor-beta [94]. This suggests a complex relationship where ATRA levels can influence the progression of cervical cancer, potentially offering pathways for therapeutic interventions.

### 4.4. Changes to Estrogen Regulation

Excessive consumption of alcohol has been linked to elevated levels of oestrone (E1), oestradiol (E2), and dehydroepiandrosterone sulfate in the bloodstream [95]. Studies have demonstrated that the steroid hormone estrogen and the presence of estrogen receptors (ERs) are linked to cancers caused by HPV infection [96]. ERs have been shown to contribute significantly to cervical cancer progression. In this context, ER antagonists such as fulvestrant and raloxifene, both of which are United States Food and Drug Administration approved for the treatment of human breast cancer, have shown promising effects in reducing cervical cancer progression in mouse models. ER antagonists in mouse models have demonstrated that these drugs can help mitigate the progression of cervical cancer and associated conditions [97]. The sex-hormone treatment can increase colony formation in HPV+ cell lines [98], while estrogen treatment can stimulate HPV16 transcript production in SiHa cells [99]. Studies on HPV+ cell lines revealed that ERα truncation, ER-α36, mediates estrogen-stimulated MAPK/ERK. Furthermore, the overexpression of this isoform resulted in enhanced invasion, migration, and proliferation of both cell lines [100]. HPV+ cervical cancer cells, such as HeLa, SiHa, and CaSki, exhibit high levels of 17β-hydroxysteroid dehydrogenase type 1 (HSD17B1), which converts estrone to estradiol. This suggests that these cells may transform the circulating hormone [101]. Additionally, alcohol consumption can increase the level of estrogen, which promotes the expression of HPV by stimulating progesterone receptors and growth factors, leading to cell proliferation [56,57].

### 4.5. Immune Suppression Compromises HPV Clearance

Alcohol consumption exerts profound immunosuppressive effects that may facilitate HPV persistence and progression to malignancy [102,103]. Multiple studies have documented that alcohol decreases the number and cytotoxic activity of natural killer (NK) cells, which play crucial roles in eliminating virus-infected and transformed cells through innate immune mechanisms [102,104]. In the context of HPV infection, reduced NK cell function may impair viral clearance and allow persistence of infection, creating conditions conducive to malignant transformation [90,102,103,105,106].

The immunosuppressive effects extend beyond NK cells to include alterations in T-cell function, cytokine production, and dendritic cell maturation [107]. Chronic alcohol exposure activates monocytes and macrophages, resulting in increased production of pro-inflammatory cytokines including tumor necrosis factor-α (TNF-α), interleukin-1 (IL-1), interleukin-6 (IL-6), and interleukin-8 (IL-8) [102]. This chronic inflammatory milieu may promote cellular transformation and support tumor progression [102,103,108].

## 5. Synergistic Effects of Tobacco and Alcohol on Cervical Cancer

Alcohol is often implicated as a social cue for smoking, and vice versa, in that people seem to smoke for prolonged periods if they consume alcohol. Studies demonstrate that individuals who use both alcohol and tobacco have significantly higher risks of developing various types of cancers than those who use either substance alone [109,110]. It is also found that the combined impact of tobacco use and alcohol consumption exceeds the multiplicative model for oral cavity cancers (OCC) [109]. Heavy alcohol consumption and smoking can lead to a 300-fold higher risk of oral and pharyngeal cancer [111]. Current smokers and alcohol consumption also increase the level of toxic and cancerous acetaldehyde in the oral cavity [112,113]. Women who consume excessive alcohol and smoke together or are exposed to secondhand smoke might be at a high risk of HPV persistence [114]. A critical milestone in causing cervical cancer. Furthermore, high alcohol intake was also associated with an increased risk of HPV infection among non-smokers and current smokers [115]. We have recently reported that Exposure to benzo[a]pyrene and/or ethanol upregulated HPV16 E6/E7 oncogene expression and epithelial–mesenchymal transition (EMT) markers in cervical cancer cells, enhancing migration and invasion. These agents activated TNF-α/NF-κB signaling, increasing IL-6 and VEGF levels, which promoted inflammation and tumor progression. Curcumin (Cur) and its PLGA nanoparticle formulation (PLGA-Cur) effectively attenuated these molecular effects, suggesting therapeutic potential in cervical cancer prevention/treatment [116].

## 6. Synergistic Effects of HPV-HIV Co-Infection and Alcohol Use on Cervical Cancer

Furthermore, high-risk sexual behaviors, including drug trading, multiple partner sex, unprotected anal intercourse, and injection drug use, can potentially lead to the acquisition of HIV and other sexually transmitted infections (STIs) [109]. Women with HIV infections are more likely to experience persistent HPV infection and cervical dysplasia progression, and vice versa. [117]. The risk of cervical cancer is six times higher in HIV-positive women than in HIV-negative women (103). HPV-HIV co-infection significantly elevates the risk of cervical cancer, and alcohol consumption may exacerbate this risk through persistent HPV infection and potentially synergistic effects with HPV viral load. Binge drinkers often exhibit high-risk HIV/HPV co-infection behaviors [118]. Binge drinkers (≥5 drinks for males or ≥4 drinks for women in the preceding 30 days) often exhibit high-risk HIV/HPV co-infection behaviors [119]. For example, binge drinkers aged 18 to 64 are twice as likely to report HIV-risky behaviors as non-binge drinkers [119]. Multiple studies have shown an association between binge drinking, unsafe sexual activities, and HIV/STI infections [120,121,122]. A study by Olusanya et al. showed that binge drinkers were more likely to engage in HIV/HPV co-infection high-risk behaviors (OR = 2.1; 95% CI: 1.0–4.5) [123].

## 7. Epidemiologic Evidence for Alcohol Consumption and Cervical Carcinogenesis

The epidemiological evidence demonstrates a consistent pattern across diverse populations, with alcohol consumption showing dose-dependent associations with HPV persistence, CIN development, and poor clinical outcomes. Most remarkably, studies have identified synergistic interactions between alcohol consumption and HPV viral load, suggesting that these two factors work in concert to dramatically amplify cervical cancer risk. The clinical implications extend beyond initial carcinogenesis to include treatment resistance and survival outcomes, indicating that alcohol’s influence persists throughout the disease continuum [12,13,103,124,125,126]. A summary of results from cohort studies of alcohol and cervical cancer is given in Table 1. A study in Sweden between 1965 and 1995 found that heavy alcohol intake increases the risk of ICC in women [12]. The findings suggested that alcoholic women had a greater incidence of in situ cervical cancer, vaginal cancer, and ICC. However, no significant increase in vulva cancer risk was observed in alcohol-consuming women. Alcoholics have an increased risk of in situ cancer, suggesting that their increased risk of ICC may not solely be due to less Pap smear screening [12]. Recently, Hy et al. found that alcohol consumption and HR-HPV load have a synergistic impact on the chance of HR-HPV persistence in Korean women, particularly for long-term infection [125]. Limiting alcohol intake might be an effective way to prevent cervical cancer in women with a high HR-HPV burden [125]. In another Korean HPV cohort study (KHPV), it was found that alcohol consumers have a higher risk of developing CIN1 (OR = 2.18, 95% CI 1.22–3.89) compared to non-drinkers, with more frequent alcohol consumption and higher ethanol consumption also linked to higher CIN1 risk. Nevertheless, no associations were found between alcohol use and CIN2/3 or cervical cancer. A recent study aimed to detect cervical neoplastic transformation in women in the alcohol abuse group, showing significantly higher levels of cellular proliferation, with Ki67 staining ranging from 29.1% to 89.7% and p16 staining ranging from 26.2% to 94.8%. The study suggests that diffuse p16 staining, the specific gravity of cells with a positive IHC response to this protein, and high reaction intensity may be used to identify CIN III and aggressive cancer in individuals with alcohol abuse [127]. A cohort of US men (N = 1313) was analyzed to evaluate the link between alcohol consumption and HPV infection prevalence among US men using data from the food frequency questionnaire (FFQ). The study found a significant increase in risk for any HPV types and oncogenic HPV types among men. The fourth quartile of alcohol consumption also increased the risk for HPV infections in all sexual partners, among never-smokers and frequent smokers, except former smokers. The study concluded that excessive alcohol consumption increases men’s risk of prevalent HPV infections [115]. Another study found that heavy alcohol use in 10% of advanced cervical cancer patients significantly decreased disease-free (HR = 10.57; 95% CI, 2.07–53.93) and overall survival (HR = 10.80; 95% CI, 2.57–45.40) rates after adjusting for covariates [13]. Studies in other populations have also shown associations between the consumption of alcohol and HPV-related outcomes. A cross-sectional study on males in the Danish army found a correlation between alcohol use and harboring numerous HPV types [128]. A prospective study on the natural history of cervicovaginal papillomavirus infection in women showed a higher risk of incident HPV infection linked to excessive alcohol intake [129]. Another cross-sectional study of sexual habits and cervical HPV infection among college women found that alcohol usage was substantially more common among women who tested positive for HPV DNA [130]. Oh et al. 2015 study found that women who recently used alcohol had a higher risk of developing continuous cervical HPV positivity for three consecutive years [OR = 2.49, 95% CI, 1.32–4.71] [124]. In contrast, other studies have shown no correlation between alcohol use and HPV outcomes [131,132].

## 8. Inconsistent Findings and Study Limitations

### 8.1. Methodological Heterogeneity

The cervical cancer literature reveals significant methodological heterogeneity that contributes to inconsistent findings across studies. Variations in alcohol exposure assessment, ranging from simple current/former drinker classifications to detailed beverage-specific consumption patterns, limit comparability between investigations. Additionally, differences in outcome definitions, with some studies focusing on invasive cancer while others examine precursor lesions, may account for apparent discrepancies in results [12,103,143,144]. Population-specific factors, including genetic susceptibility, cultural drinking patterns, and healthcare access, likely contribute to geographic variations in observed associations. The robust associations documented in Korean populations may reflect genetic polymorphisms in alcohol-metabolizing enzymes that are less prevalent in European or North American populations [90,145].

### 8.2. Sample Size and Statistical Power

Many studies examining alcohol-cervical cancer associations suffer from inadequate statistical power, particularly for detecting modest increases in risk for relatively uncommon outcomes. The Duke University pilot study, for example, included only 35 participants and found no association between harmful alcohol use and high-grade dysplasia, but the confidence intervals were extremely wide, indicating insufficient precision for meaningful conclusions [144]. Similarly, the Los Angeles CHIS analysis, despite finding significant associations, included only 441 women with both alcohol consumption and cervical cancer data, limiting generalizability and precision of risk estimates. These sample size limitations highlight the need for larger, well-designed studies to definitively establish the magnitude of alcohol-associated cervical cancer risk [143].

### 8.3. Confounding and Bias Considerations

Epidemiological studies of alcohol and cervical cancer face significant challenges related to confounding by associated lifestyle factors and potential bias in exposure assessment. Sexual behavior, which is strongly associated with both alcohol consumption and HPV acquisition, is often inadequately assessed in health screening questionnaires due to cultural sensitivities. This limitation is particularly problematic in Asian populations, where detailed sexual history collection may be culturally challenging [103,124]. Recall bias in alcohol consumption assessment represents another significant limitation, as retrospective exposure assessment may be influenced by disease status or social desirability factors. Prospective cohort studies with detailed alcohol exposure assessment collected before disease development provide the most reliable evidence, but such studies remain relatively uncommon in the cervical cancer literature [12,103,124].

## 9. Clinical Implications and Public Health Perspectives

### 9.1. Screening and Prevention Strategies

The emerging evidence for alcohol-cervical cancer associations has important implications for clinical practice and public health policy. Women with significant alcohol consumption histories may benefit from enhanced cervical cancer surveillance, particularly in the presence of persistent HPV infection. The synergistic interaction between alcohol and HPV suggests that alcohol cessation interventions could potentially reduce cervical cancer risk among HPV-positive women [103,124]. Healthcare providers should consider alcohol consumption patterns when counseling women about cervical cancer risk factors, particularly in populations with high prevalence of alcohol-metabolizing enzyme polymorphisms. The demonstration that even light-to-moderate alcohol consumption may increase HPV persistence risk suggests that current “safe” drinking guidelines may not apply to cervical cancer prevention [90,124,145].

### 9.2. Integration with HPV Vaccination Programs

The alcohol-HPV interactions documented in recent studies have implications for HPV vaccination program design and implementation. While HPV vaccination remains the most effective primary prevention strategy for cervical cancer, the potential for alcohol to enhance carcinogenesis in unvaccinated populations or in the context of non-vaccine HPV types should inform comprehensive prevention approaches [146,147]. Countries implementing cervical cancer elimination strategies should consider alcohol consumption patterns in their populations when designing screening and vaccination programs. The World Health Organization’s goal of cervical cancer elimination through 90% HPV vaccination coverage, 70% screening participation, and 90% treatment of identified lesions may require modification in populations with high alcohol consumption prevalence [148,149].

### 9.3. Treatment Considerations

Evidence suggests that heavy alcohol consumption may negatively impact cervical cancer treatment outcomes. A retrospective study of locally advanced cervical cancer patients found that alcohol consumption was significantly associated with decreased disease-free survival, overall survival, and increased risk for pelvic recurrence. These findings suggest that alcohol cessation should be integrated into comprehensive cervical cancer treatment protocols [13]. The mechanisms underlying poor treatment outcomes in alcohol-consuming cervical cancer patients likely involve the same immunosuppressive and oxidative stress pathways that contribute to initial carcinogenesis. Addressing alcohol consumption as part of survivorship care may improve long-term outcomes and reduce recurrence risk [13,90].

## 10. Future Research Directions and Recommendations

### 10.1. Mechanistic Studies

Future research should prioritize detailed mechanistic investigations to elucidate the biological pathways linking alcohol consumption to cervical carcinogenesis. Studies examining the effects of alcohol metabolites on HPV integration, oncogene expression, and host immune responses will provide crucial insights for prevention and treatment strategies. Additionally, research into genetic susceptibility factors, particularly alcohol-metabolizing enzyme polymorphisms, will help identify high-risk populations for targeted interventions [90,145].

### 10.2. Large-Scale Prospective Cohorts

The field requires large-scale, prospective cohort studies with detailed alcohol exposure assessment, HPV genotyping, and long-term follow-up to definitively establish the magnitude and mechanisms of alcohol-associated cervical cancer risk. Such studies should include diverse populations to assess generalizability across different genetic and cultural backgrounds [12,124]. Standardized exposure assessment protocols, including validated questionnaires for alcohol consumption patterns and biomarker validation of self-reported consumption, will improve comparability between studies and reduce measurement error. Integration of molecular epidemiology approaches, including HPV genotyping, viral load assessment, and host genetic profiling, will provide comprehensive insights into alcohol-HPV interactions [103,124,125,143,144].

### 10.3. Intervention Studies

Randomized controlled trials examining the effects of alcohol cessation interventions on HPV clearance and cervical lesion regression will provide the strongest evidence for causal relationships and inform clinical practice guidelines. Such studies are ethically feasible given the established health benefits of alcohol cessation and could provide definitive evidence for alcohol’s role in cervical carcinogenesis [103,124].

## 11. Conclusions

The accumulated epidemiological evidence supports a modest but consistent association between alcohol consumption and increased risk of cervical precursor lesions, particularly in the context of high-risk HPV infection. The synergistic interaction between alcohol consumption and HPV viral load, demonstrated most convincingly in Korean population studies, provides compelling evidence for biological causation rather than simple confounding by associated lifestyle factors. The temporal relationship between alcohol exposure and early cervical lesions, combined with dose–response patterns across consumption frequency and quantity, further supports a causal interpretation [103,124,125].

However, in the literature, particularly regarding associations with advanced cervical lesions and invasive cervical cancer, these inconsistencies may reflect genuine biological differences in alcohol’s effects on early versus advanced carcinogenesis, methodological limitations, including inadequate statistical power, or population-specific factors, including genetic susceptibility variations [90,103,145,146].

The biological mechanisms linking alcohol to cervical carcinogenesis are well-established and include direct DNA damage through acetaldehyde formation, immune system suppression affecting HPV clearance, oxidative stress induction, and metabolic effects on folate and estrogen pathways. These mechanisms provide strong biological plausibility for observed epidemiological associations [90,108].

From a public health perspective, the evidence is sufficient to warrant inclusion of alcohol consumption in cervical cancer risk factor counseling, particularly for women with persistent HPV infection. Healthcare providers should consider alcohol consumption patterns when developing individualized screening and prevention strategies. However, the evidence is not yet sufficient to establish specific alcohol consumption thresholds for cervical cancer risk or to recommend alcohol cessation as a primary prevention strategy in the general population [103,124].

The time has come to draw preliminary conclusions while acknowledging remaining uncertainties. Alcohol consumption appears to act as a cofactor in HPV-mediated cervical carcinogenesis, with the strongest evidence supporting effects on the transition from transient to persistent HPV infection and the development of early cervical lesions. This conclusion is based on consistent findings across multiple high-quality studies, demonstration of biological interactions with HPV, and well-established carcinogenic mechanisms [90,103,108,124,125].

Future research should focus on large-scale prospective studies with comprehensive exposure assessment and molecular epidemiology approaches to establish causal relationships and inform evidence-based prevention strategies. Until such studies are completed, a precautionary approach emphasizing alcohol moderation as part of comprehensive cervical cancer prevention appears justified based on current evidence [12,90,103,124].

## Figures and Tables

**Table 1:** Summary of epidemiologic insights for alcohol consumption and cervical carcinogenesis.

Research Title (Publishing Year)	Study Type	Country (Study Period)	Cases/Study Size	Alcohol Consumption	RR (95% CI)	References
Global distribution, risk factors, and recent trends for cervical cancer: A worldwide country-level analysis (2022)	GLOBOCAN database	Globally in 2018	311,365 deaths/568,847 cases	Alcohol consumption	β = 1.89, 95% CI = 0.59–3.19, *p* = 0.005; β = 0.98, CI = 0.08–1.88, *p* = 0.033	[133]
Factors associated with normal or abnormal Papanicolaou smear among HIV women at a national hospital in Lima, Peru, 2012–2015 (2021)	Case–control	Lima, Peru (2012–2015)	368 patients	Alcohol consumption	Abnormal Pap smear OR, 1.77; 95% CI = 1.06–2.95	[134]
Cervical cancer and precancerous cervical lesions detected using visual inspection with acetic acid at Livingstone Teaching Hospital (2021)	Cross-sectional	Zambia (2019–2020)	329 women’s	Alcohol consumption	OR, 0.30; 95% CI = 0.12–0.74	[135]
Prevalence of sexually transmitted infections among cervical cancer suspected women at University of Gondar Comprehensive	Cross-sectional	Ethiopia (February–April 2017)	403 women’s	Alcohol addiction	OR, 2.2; 95% CI = 1.07–4.5, *p* = 0.031	[136]
Specialized Hospital, North-west Ethiopia (2021)						
Risk of cancer in individuals with alcohol and drug use disorders: a registry-based study in Reggio Emilia, Italy (2020)	Population-based cohort	Italy (1996–2014)	4373 patients	Alcohol use	SIR = 8.6; 95% CI = 2.8–26.7	[137]
Alcohol Abuse Decreases Pelvic Control and Survival in Cervical Cancer: An Opportunity of Lifestyle Intervention for Outcome Improvement (2017)	Retrospective	USA (2007–2013)	95 patients	Alcohol abuse decreases the DFS	*p* = 0.005; HR, 10.57; 95% CI = 2.07–53.93 and OS *p* = 0.001; HR, 10.80; 95% CI: 2.57–45.40.	[13]
Synergistic effect of viral load and alcohol consumption on the risk of persistent high-risk human papillomavirus infection (2014)	Prospective cohort	Korea (2002–2011)	11,140 patients	Diagnosis of alcohol	HR-HPV, 1 year follow-up OR, 4.14; 95% CI = 1.89–9.05; 2-year persistence (OR, 6.61; 95% CI = 2.09–20.9)	[125].
Alcohol consumption and viral load are synergistically associated with CIN1 (2013)	Cohort	Korea (2006–2009)	1243 patients	Alcohol drinker	Increased risk of CINI; OR, 2.18; 95% CI = 1.22–3.89; High HPV viral load (≥100 RLU/PC) with high risk of CINI, OR, 19.1; 95% CI= 6.60–55.3	[103]
Moderate alcohol intake and cancer incidence in women (2009)	Prospective cohort	United Kingdom (1996–2001)	1,280,296 middle-aged women	Women drinking several drinks/week	RR, 95% floated CI varies across different sample sizes.	[138]
Alcoholism and risk for cancer of the cervix uteri, vagina, and vulva (2001)	Population-based cohort	Sweden (1965–1995)	36,856 patients	Diagnosis of alcoholism	In situ cervical cancer (SIR, 1.7; 95% CI = 1.6–1.9, Invasive cervical cancer SIR, 2.9; 95% CI = 2.4–3.5); Cancer of vagina (SIR, 4 4.6; 95% CI = 2.2–8.5)	[12]
Increased cancer risk among Swedish female alcoholics (1996)	Population-based cohort	Sweden (1917–1977)	187/15,508 alcoholic women’s	Alcohol abuse	Cervix uteri, RR, 3.9; 95% CI = 2.8–5.4 vulva, vagina, and unspecified female genital organs (RR,4.0; 95% CI = 1.3–12)	[139]
Cancer incidence among waitresses in Norway (1994)	Population-based cohort	Norway (1959–1991)	5314 waitresses (21/38 cases)	Alcohol consumption	SIR, 1.8; 95% CI	[140]
Cancer morbidity in alcohol abusers (1994)	Population-based cohort	Denmark (1954–1987)	22/3093	Alcohol consumption	RR, 2.0; 95% CI = 1.2–3.0	[141]
Alcoholism and cancer risk: a population-based cohort study (1992)	Population-based cohort	Sweden (1965–1983)	6/1013	Diagnosis of alcohol	SIR, 4.2; 95% CI = 1.5–9.1	[142]
Natural history of cervicovaginal papillomavirus infection in young women, 1998	Prospective study	State University in New Brunswick, New Jersey	608 female students	High alcohol consumption	RR, 2.0; 95% CI = 1.2–3.1	[129]

## Data Availability

Not applicable.

## References

[1] Siegel R.L., Giaquinto A.N., Jemal A. (2024). Cancer statistics, 2024. CA Cancer J. Clin..

[2] Kashyap V.K., Dan N., Chauhan N., Wang Q., Setua S., Nagesh P.K.B., Malik S., Batra V., Yallapu M.M., Miller D.D. (2020). VERU-111 suppresses tumor growth and metastatic phenotypes of cervical cancer cells through the activation of p53 signaling pathway. Cancer Lett..

[3] Karuri A.R., Kashyap V.K., Yallapu M.M., Zafar N., Kedia S.K., Jaggi M., Chauhan S.C. (2017). Disparity in rates of HPV infection and cervical cancer in underserved US populations. Front. Biosci. (Schol. Ed.).

[4] Li M., Wang J., Geng Y., Li Y., Wang Q., Liang Q., Qi Q. (2012). A strategy of gene overexpression based on tandem repetitive promoters in Escherichia coli. Microb. Cell Fact..

[5] Islami F., Goding Sauer A., Miller K.D., Siegel R.L., Fedewa S.A., Jacobs E.J., McCullough M.L., Patel A.V., Ma J., Soerjomataram I. (2018). Proportion and number of cancer cases and deaths attributable to potentially modifiable risk factors in the United States. CA Cancer J. Clin..

[6] Connor J. (2017). Alcohol consumption as a cause of cancer. Addiction.

[7] International Agency for Research on Cancer (IARC) (1988). Alcohol Drinking. IARC Monographs on the Evaluation of Carcinogenic Risks to Humans.

[8] Alcohol and Cancer Risk: The U.S. (2025). Surgeon General’s Advisory. https://www.hhs.gov/surgeongeneral/reports-and-publications/addiction-and-substance-misuse/index.html.

[9] Seitz H.K., Becker P. (2007). Alcohol metabolism and cancer risk. Alcohol. Res. Health.

[10] Dinis-Oliveira R.J. (2016). Oxidative and Non-Oxidative Metabolomics of Ethanol. Curr. Drug Metab..

[11] Shukla S.D., Lim R.W. (2013). Epigenetic effects of ethanol on the liver and gastrointestinal system. Alcohol. Res..

[12] Weiderpass E., Ye W., Tamimi R., Trichopolous D., Nyren O., Vainio H., Adami H.O. (2001). Alcoholism and risk for cancer of the cervix uteri, vagina, and vulva. Cancer Epidemiol. Biomark. Prev..

[13] Mayadev J., Li C.S., Lim J., Valicenti R., Alvarez E.A. (2017). Alcohol Abuse Decreases Pelvic Control and Survival in Cervical Cancer: An Opportunity of Lifestyle Intervention for Outcome Improvement. Am. J. Clin. Oncol..

[14] Praud D., Rota M., Rehm J., Shield K., Zatoński W., Hashibe M., La Vecchia C., Boffetta P. (2016). Cancer incidence and mortality attributable to alcohol consumption. Int. J. Cancer.

[15] Nelson D.E., Jarman D.W., Rehm J., Greenfield T.K., Rey G., Kerr W.C., Miller P., Shield K.D., Ye Y., Naimi T.S. (2013). Alcohol-attributable cancer deaths and years of potential life lost in the United States. Am. J. Public. Health.

[16] Rumgay H., Shield K., Charvat H., Ferrari P., Sornpaisarn B., Obot I., Islami F., Lemmens V., Rehm J., Soerjomataram I. (2021). Global burden of cancer in 2020 attributable to alcohol consumption: A population-based study. Lancet Oncol..

[17] Esser M.B., Sherk A., Liu Y., Naimi T.S. (2024). Deaths from Excessive Alcohol Use—United States, 2016–2202. MMWR Morb. Mortal. Wkly. Rep..

[18] Alcohol and Cancer Risk. https://www.cancer.gov/about-cancer/causes-prevention/risk/alcohol/alcohol-fact-sheet#r4.

[19] Bagnardi V., Rota M., Botteri E., Tramacere I., Islami F., Fedirko V., Scotti L., Jenab M., Turati F., Pasquali E. (2015). Alcohol consumption and site-specific cancer risk: A comprehensive dose-response meta-analysis. Br. J. Cancer.

[20] Zhao J., Stockwell T., Roemer A., Chikritzhs T. (2016). Is alcohol consumption a risk factor for prostate cancer? A systematic review and meta-analysis. BMC Cancer.

[21] Moore A.A., Gould R., Reuben D.B., Greendale G.A., Carter M.K., Zhou K., Karlamangla A. (2005). Longitudinal patterns and predictors of alcohol consumption in the United States. Am. J. Public Health.

[22] Breslow R.A., Graubard B.I. (2008). Prospective study of alcohol consumption in the United States: Quantity, frequency, and cause-specific mortality. Alcohol. Clin. Exp. Res..

[23] Edelman E.J., Fiellin D.A. (2016). In the Clinic. Alcohol Use. Ann. Intern. Med..

[24] WHO (2023). No Level of Alcohol Consumption is Safe for Our Health. 4 January 2023.

[25] Hingson R.W., Zha W., White A.M. (2017). Drinking Beyond the Binge Threshold: Predictors, Consequences, and Changes in the U.S. Am. J. Prev. Med..

[26] Esser M.B., Hedden S.L., Kanny D., Brewer R.D., Gfroerer J.C., Naimi T.S. (2014). Prevalence of alcohol dependence among US adult drinkers, 2009–2011. Prev. Chronic Dis..

[27] CDC (2023). Alcohol Use and Your Health.

[28] Molina P.E., Happel K.I., Zhang P., Kolls J.K., Nelson S. (2010). Focus on: Alcohol and the immune system. Alcohol. Res. Health.

[29] Li J., Li S. (2025). From Viral Infection to Genome Reshaping: The Triggering Role of HPV Integration in Cervical Cancer. Int. J. Mol. Sci..

[30] de Sanjosé S., Diaz M., Castellsagué X., Clifford G., Bruni L., Muñoz N., Bosch F.X. (2007). Worldwide prevalence and genotype distribution of cervical human papillomavirus DNA in women with normal cytology: A meta-analysis. Lancet Infect. Dis..

[31] Bao Y.P., Li N., Smith J.S., Qiao Y.L. (2008). Human papillomavirus type distribution in women from Asia: A meta-analysis. Int. J. Gynecol. Cancer.

[32] Eckert L.O.N., Moscicki A.B. (2017). Committee Opinion No. 704: Human Papillomavirus Vaccination. Obstet. Gynecol..

[33] Adam E., Berkova Z., Daxnerova Z., Icenogle J., Reeves W.C., Kaufman R.H. (2000). Papillomavirus detection: Demographic and behavioral characteristics influencing the identification of cervical disease. Am. J. Obstet. Gynecol..

[34] Lehtinen M., Koskela P., Jellum E., Bloigu A., Anttila T., Hallmans G., Luukkaala T., Thoresen S., Youngman L., Dillner J. (2002). Herpes simplex virus and risk of cervical cancer: A longitudinal, nested case-control study in the nordic countries. Am. J. Epidemiol..

[35] Muñoz N., Franceschi S., Bosetti C., Moreno V., Herrero R., Smith J.S., Shah K.V., Meijer C.J., Bosch F.X. (2002). Role of parity and human papillomavirus in cervical cancer: The IARC multicentric case-control study. Lancet.

[36] Urbute A., Frederiksen K., Thomsen L.T., Kesmodel U.S., Kjaer S.K. (2024). Overweight and obesity as risk factors for cervical cancer and detection of precancers among screened women: A nationwide, population-based cohort study. Gynecol. Oncol..

[37] Sugawara Y., Tsuji I., Mizoue T., Inoue M., Sawada N., Matsuo K., Ito H., Naito M., Nagata C., Kitamura Y. (2018). Cigarette smoking and cervical cancer risk: An evaluation based on a systematic review and meta-analysis among Japanese women. Jpn. J. Clin. Oncol..

[38] Collins S., Rollason T.P., Young L.S., Woodman C.B. (2010). Cigarette smoking is an independent risk factor for cervical intraepithelial neoplasia in young women: A longitudinal study. Eur. J. Cancer.

[39] Louie K.S., Castellsague X., de Sanjose S., Herrero R., Meijer C.J., Shah K., Munoz N., Bosch F.X. (2011). Smoking and passive smoking in cervical cancer risk: Pooled analysis of couples from the IARC multicentric case-control studies. Cancer Epidemiol. Biomark. Prev..

[40] Aguayo F., Muñoz J.P., Perez-Dominguez F., Carrillo-Beltrán D., Oliva C., Calaf G.M., Blanco R., Nuñez-Acurio D. (2020). High-Risk Human Papillomavirus and Tobacco Smoke Interactions in Epithelial Carcinogenesis. Cancers.

[41] Burk R.D., Kelly P., Feldman J., Bromberg J., Vermund S.H., DeHovitz J.A., Landesman S.H. (1996). Declining prevalence of cervicovaginal human papillomavirus infection with age is independent of other risk factors. Sex. Transm. Dis..

[42] Okunade K.S. (2020). Human papillomavirus and cervical cancer. J. Obstet. Gynaecol..

[43] Unger E.R., Barr E. (2004). Human papillomavirus and cervical cancer. Emerg. Infect. Dis..

[44] Walboomers J.M., Jacobs M.V., Manos M.M., Bosch F.X., Kummer J.A., Shah K.V., Snijders P.J., Peto J., Meijer C.J., Muñoz N. (1999). Human papillomavirus is a necessary cause of invasive cervical cancer worldwide. J. Pathol..

[45] DiGiuseppe S., Bienkowska-Haba M., Guion L.G.M., Keiffer T.R., Sapp M. (2017). Human Papillomavirus Major Capsid Protein L1 Remains Associated with the Incoming Viral Genome throughout the Entry Process. J. Virol..

[46] Stanley M.A., Pett M.R., Coleman N. (2007). HPV: From infection to cancer. Biochem. Soc. Trans..

[47] Apt D., Watts R.M., Suske G., Bernard H.U. (1996). High Sp1/Sp3 ratios in epithelial cells during epithelial differentiation and cellular transformation correlate with the activation of the HPV-16 promoter. Virology.

[48] Cripe T.P., Alderborn A., Anderson R.D., Parkkinen S., Bergman P., Haugen T.H., Pettersson U., Turek L.P. (1990). Transcriptional activation of the human papillomavirus-16 P97 promoter by an 88-nucleotide enhancer containing distinct cell-dependent and AP-1-responsive modules. New Biol..

[49] Gravitt P.E., Winer R.L. (2017). Natural History of HPV Infection across the Lifespan: Role of Viral Latency. Viruses.

[50] Doorbar J. (2005). The papillomavirus life cycle. J. Clin. Virol. Off. Publ. Pan Am. Soc. Clin. Virol..

[51] Burd E.M. (2003). Human papillomavirus and cervical cancer. Clin. Microbiol. Rev..

[52] Pal A., Kundu R. (2019). Human Papillomavirus E6 and E7: The Cervical Cancer Hallmarks and Targets for Therapy. Front. Microbiol..

[53] Moody C.A., Laimins L.A. (2010). Human papillomavirus oncoproteins: Pathways to transformation. Nat. Rev. Cancer.

[54] Li S., Hong X., Wei Z., Xie M., Li W., Liu G., Guo H., Yang J., Wei W., Zhang S. (2019). Ubiquitination of the HPV Oncoprotein E6 Is Critical for E6/E6AP-Mediated p53 Degradation. Front. Microbiol..

[55] Rasi Bonab F., Baghbanzadeh A., Ghaseminia M., Bolandi N., Mokhtarzadeh A., Amini M., Dadashzadeh K., Hajiasgharzadeh K., Baradaran B., Bannazadeh Baghi H. (2021). Molecular pathways in the development of HPV-induced cervical cancer. Excli J..

[56] Auborn K.J., Woodworth C., DiPaolo J.A., Bradlow H.L. (1991). The interaction between HPV infection and estrogen metabolism in cervical carcinogenesis. Int. J. Cancer.

[57] Espina N., Lima V., Lieber C.S., Garro A.J. (1988). In vitro and in vivo inhibitory effect of ethanol and acetaldehyde on O6-methylguanine transferase. Carcinogenesis.

[58] Seitz H.K., Stickel F. (2007). Molecular mechanisms of alcohol-mediated carcinogenesis. Nat. Rev. Cancer.

[59] Mizumoto A., Ohashi S., Hirohashi K., Amanuma Y., Matsuda T., Muto M. (2017). Molecular Mechanisms of Acetaldehyde-Mediated Carcinogenesis in Squamous Epithelium. Int. J. Mol. Sci..

[60] Varela-Rey M., Woodhoo A., Martinez-Chantar M.L., Mato J.M., Lu S.C. (2013). Alcohol, DNA methylation, and cancer. Alcohol. Res..

[61] Seitz H.K., Stickel F. (2010). Acetaldehyde as an underestimated risk factor for cancer development: Role of genetics in ethanol metabolism. Genes Nutr..

[62] Han X., Fang F., Cui W., Liu Y., Liu Y. (2023). Effect of Ethanol-Induced Methyl Donors Consumption on the State of Hypomethylation in Cervical Cancer. Int. J. Mol. Sci..

[63] Oze I., Matsuo K., Suzuki T., Kawase T., Watanabe M., Hiraki A., Ito H., Hosono S., Ozawa T., Hatooka S. (2009). Impact of multiple alcohol dehydrogenase gene polymorphisms on risk of upper aerodigestive tract cancers in a Japanese population. Cancer Epidemiol. Biomark. Prev..

[64] Imani M.M., Moradi M.M., Rezaei F., Mozaffari H.R., Sharifi R., Safaei M., Azizi F., Basamtabar M., Sohrabi Z., Shalchi M. (2024). Association between alcohol dehydrogenase polymorphisms (rs1229984, rs1573496, rs1154460, and rs284787) and susceptibility to head and neck cancers: A systematic review and meta-analysis. Arch. Oral Biol..

[65] Chang T.G., Yen T.T., Wei C.Y., Hsiao T.H., Chen I.C. (2023). Impacts of ADH1B rs1229984 and ALDH2 rs671 polymorphisms on risks of alcohol-related disorder and cancer. Cancer Med..

[66] Chen C.C., Lu R.B., Chen Y.C., Wang M.F., Chang Y.C., Li T.K., Yin S.J. (1999). Interaction between the functional polymorphisms of the alcohol-metabolism genes in protection against alcoholism. Am. J. Hum. Genet..

[67] Coutelle C., Höhn B., Benesova M., Oneta C.M., Quattrochi P., Roth H.J., Schmidt-Gayk H., Schneeweiss A., Bastert G., Seitz H.K. (2004). Risk factors in alcohol associated breast cancer: Alcohol dehydrogenase polymorphism and estrogens. Int. J. Oncol..

[68] Alzahrani A.M., Rajendran P. (2020). The Multifarious Link between Cytochrome P450s and Cancer. Oxid. Med. Cell. Longev..

[69] Sakamoto T., Hara M., Higaki Y., Ichiba M., Horita M., Mizuta T., Eguchi Y., Yasutake T., Ozaki I., Yamamoto K. (2006). Influence of alcohol consumption and gene polymorphisms of ADH2 and ALDH2 on hepatocellular carcinoma in a Japanese population. Int. J. Cancer.

[70] Hayashi S., Watanabe J., Kawajiri K. (1991). Genetic polymorphisms in the 5’-flanking region change transcriptional regulation of the human cytochrome P450IIE1 gene. J. Biochem..

[71] Persson I., Johansson I., Bergling H., Dahl M.L., Seidegård J., Rylander R., Rannug A., Högberg J., Sundberg M.I. (1993). Genetic polymorphism of cytochrome P4502E1 in a Swedish population. Relationship to incidence of lung cancer. FEBS Lett..

[72] Liu W., Tian F., Dai L., Chai Y. (2014). Cytochrome P450 2E1 gene polymorphism and alcohol drinking on the risk of hepatocellular carcinoma: A meta-analysis. Mol. Biol. Rep..

[73] Orywal K., Jelski W., Zdrodowski M., Szmitkowski M. (2016). The Diagnostic Value of Alcohol Dehydrogenase Isoenzymes and Aldehyde Dehydrogenase Measurement Sera of Cervical Cancer Patients. Anticancer Res..

[74] Orywal K., Jelski W., Zdrodowski M., Szmitkowski M. (2011). The activity of class I, II, III and IV alcohol dehydrogenase isoenzymes and aldehyde dehydrogenase in cervical cancer. Clin. Biochem..

[75] Orywal K., Jelski W., Zdrodowski M., Szmitkowski M. (2013). The diagnostic value of alcohol dehydrogenase isoenzymes and aldehyde dehydrogenase measurement in the sera of patients with endometrial cancer. Anticancer Res..

[76] Seitz H.K., Stickel F. (2006). Risk factors and mechanisms of hepatocarcinogenesis with special emphasis on alcohol and oxidative stress. Biol. Chem..

[77] Letafati A., Taghiabadi Z., Zafarian N., Tajdini R., Mondeali M., Aboofazeli A., Chichiarelli S., Saso L., Jazayeri S.M. (2024). Emerging paradigms: Unmasking the role of oxidative stress in HPV-induced carcinogenesis. Infect. Agent. Cancer.

[78] Wu W.S. (2006). The signaling mechanism of ROS in tumor progression. Cancer Metastasis Rev..

[79] Despot A., Fureš R., Despot A.M., Mikuš M., Zlopaša G., D’Amato A., Chiantera V., Serra P., Etrusco A., Laganà A.S. (2023). Reactive oxygen species within the vaginal space: An additional promoter of cervical intraepithelial neoplasia and uterine cervical cancer development?. Open Med..

[80] Aleynik S.I., Leo M.A., Aleynik M.K., Lieber C.S. (1998). Increased circulating products of lipid peroxidation in patients with alcoholic liver disease. Alcohol. Clin. Exp. Res..

[81] el Ghissassi F., Barbin A., Nair J., Bartsch H. (1995). Formation of 1,N6-ethenoadenine and 3,N4-ethenocytosine by lipid peroxidation products and nucleic acid bases. Chem. Res. Toxicol..

[82] Haorah J., Ramirez S.H., Floreani N., Gorantla S., Morsey B., Persidsky Y. (2008). Mechanism of alcohol-induced oxidative stress and neuronal injury. Free Radic. Biol. Med..

[83] Hu W., Feng Z., Eveleigh J., Iyer G., Pan J., Amin S., Chung F.L., Tang M.S. (2002). The major lipid peroxidation product, trans-4-hydroxy-2-nonenal, preferentially forms DNA adducts at codon 249 of human p53 gene, a unique mutational hotspot in hepatocellular carcinoma. Carcinogenesis.

[84] Shinohara M., Adachi Y., Mitsushita J., Kuwabara M., Nagasawa A., Harada S., Furuta S., Zhang Y., Seheli K., Miyazaki H. (2010). Reactive oxygen generated by NADPH oxidase 1 (Nox1) contributes to cell invasion by regulating matrix metalloprotease-9 production and cell migration. J. Biol. Chem..

[85] Kwaśniewska A., Goździcka-Józefiak A., Borzecki A., Baranowski W. (2004). DNA adducts in squamous cell cervical carcinomas associated with HPV infection. Eur. J. Gynaecol. Oncol..

[86] Villéger R., Chulkina M., Mifflin R.C., Powell D.W., Pinchuk I.V. (2023). Disruption of retinol-mediated IL-6 expression in colon cancer-associated fibroblasts: New perspectives on the role of vitamin A metabolism. Oncotarget.

[87] Helm C.W., Lorenz D.J., Meyer N.J., Rising W.W., Wulff J.L. (2013). Retinoids for preventing the progression of cervical intra-epithelial neoplasia. Cochrane Database Syst. Rev..

[88] Choo C.K., Rorke E.A., Eckert R.L. (1995). Retinoid regulation of cell differentiation in a series of human papillomavirus type 16-immortalized human cervical epithelial cell lines. Carcinogenesis.

[89] Wang X.D. (2003). Retinoids and alcohol-related carcinogenesis. J. Nutr..

[90] Ratna A., Mandrekar P. (2017). Alcohol and Cancer: Mechanisms and Therapies. Biomolecules.

[91] Duester G. (1998). Alcohol dehydrogenase as a critical mediator of retinoic acid synthesis from vitamin A in the mouse embryo. J. Nutr..

[92] Chute J.P., Muramoto G.G., Whitesides J., Colvin M., Safi R., Chao N.J., McDonnell D.P. (2006). Inhibition of aldehyde dehydrogenase and retinoid signaling induces the expansion of human hematopoietic stem cells. Proc. Natl. Acad. Sci. USA.

[93] Berlin Grace V.M., Niranjali Devaraj S., Radhakrishnan Pillai M., Devaraj H. (2006). HPV-induced carcinogenesis of the uterine cervix is associated with reduced serum ATRA level. Gynecol. Oncol..

[94] Behbakht K., DeGeest K., Turyk M.E., Wilbanks G.D. (1996). All-trans-retinoic acid inhibits the proliferation of cell lines derived from human cervical neoplasia. Gynecol. Oncol..

[95] Liu Y., Nguyen N., Colditz G.A. (2015). Links between alcohol consumption and breast cancer: A look at the evidence. Womens Health.

[96] James C.D., Morgan I.M., Bristol M.L. (2020). The Relationship between Estrogen-Related Signaling and Human Papillomavirus Positive Cancers. Pathogens.

[97] Chung S.H., Franceschi S., Lambert P.F. (2010). Estrogen and ERalpha: Culprits in cervical cancer?. Trends Endocrinol. Metab. TEM.

[98] Hellberg D. (2012). Sex steroids and cervical cancer. Anticancer Res..

[99] Mitrani-Rosenbaum S., Tsvieli R., Tur-Kaspa R. (1989). Oestrogen stimulates differential transcription of human papillomavirus type 16 in SiHa cervical carcinoma cells. J. Gen. Virol..

[100] Sun Q., Liang Y., Zhang T., Wang K., Yang X. (2017). ER-α36 mediates estrogen-stimulated MAPK/ERK activation and regulates migration, invasion, proliferation in cervical cancer cells. Biochem. Biophys. Res. Commun..

[101] Tomaszewska A., Roszak A., Pawlik P., Sajdak S., Jagodziński P.P. (2015). Increased 17ß-hydroxysteroid dehydrogenase type 1 levels in primary cervical cancer. Biomed. Pharmacother..

[102] Meadows G.G., Zhang H. (2015). Effects of Alcohol on Tumor Growth, Metastasis, Immune Response, and Host Survival. Alcohol. Res..

[103] Min K.-J., Lee J.-K., Lee S., Kim M.K. (2013). Alcohol Consumption and Viral Load Are Synergistically Associated with CIN1. PLoS ONE.

[104] Ben-Eliyahu S., Page G.G., Yirmiya R., Taylor A.N. (1996). Acute alcohol intoxication suppresses natural killer cell activity and promotes tumor metastasis. Nat. Med..

[105] Laso F.J., Madruga J.I., Girón J.A., López A., Ciudad J., San Miguel J.F., Alvarez-Mon M., Orfao A. (1997). Decreased natural killer cytotoxic activity in chronic alcoholism is associated with alcohol liver disease but not active ethanol consumption. Hepatology.

[106] Wu X., Xiao Y., Guo D., Zhang Z., Liu M. (2022). Reduced NK Cell Cytotoxicity by Papillomatosis-Derived TGF-β Contributing to Low-Risk HPV Persistence in JORRP Patients. Front. Immunol..

[107] Zhang Z., Liu M., An Y., Gao C., Wang T., Zhang Z., Zhang G., Li S., Li W., Li M. (2025). Targeting immune microenvironment in cervical cancer: Current research and advances. J. Transl. Med..

[108] Rumgay H., Murphy N., Ferrari P., Soerjomataram I. (2021). Alcohol and Cancer: Epidemiology and Biological Mechanisms. Nutrients.

[109] Turati F., Garavello W., Tramacere I., Pelucchi C., Galeone C., Bagnardi V., Corrao G., Islami F., Fedirko V., Boffetta P. (2013). A meta-analysis of alcohol drinking and oral and pharyngeal cancers: Results from subgroup analyses. Alcohol Alcohol..

[110] Pelucchi C., Gallus S., Garavello W., Bosetti C., La Vecchia C. (2006). Cancer risk associated with alcohol and tobacco use: Focus on upper aero-digestive tract and liver. Alcohol Res. Health J. Natl. Inst. Alcohol Abus. Alcohol..

[111] Boyle P. (2010). Tobacco: Science, Policy and Public Health.

[112] Salaspuro V., Salaspuro M. (2004). Synergistic effect of alcohol drinking and smoking on in vivo acetaldehyde concentration in saliva. Int. J. Cancer.

[113] Oki E., Zhao Y., Yoshida R., Egashira A., Ohgaki K., Morita M., Kakeji Y., Maehara Y. (2009). The difference in p53 mutations between cancers of the upper and lower gastrointestinal tract. Digestion.

[114] Seo S.-S., Oh H.Y., Kim M.K., Lee D.O., Chung Y.K., Kim J.-Y., Lee C.W. (2019). Combined Effect of Secondhand Smoking and Alcohol Drinking on Risk of Persistent Human Papillomavirus Infection. BioMed Res. Int..

[115] Schabath M.B., Thompson Z.J., Egan K.M., Torres B.N., Nguyen A., Papenfuss M.R., Abrahamsen M.E., Giuliano A.R. (2015). Alcohol consumption and prevalence of human papillomavirus (HPV) infection among US men in the HPV in Men (HIM) study. Sex. Transm. Infect..

[116] Kashyap V.K., Nagesh P.K.B., Singh A.K., Massey A., Darkwah G.P., George A., Khan S., Hafeez B.B., Zafar N., Kumar S. (2024). Curcumin attenuates smoking and drinking activated NF-κB/IL-6 inflammatory signaling axis in cervical cancer. Cancer Cell Int..

[117] Houlihan C.F., Larke N.L., Watson-Jones D., Smith-McCune K.K., Shiboski S., Gravitt P.E., Smith J.S., Kuhn L., Wang C., Hayes R. (2012). Human papillomavirus infection and increased risk of HIV acquisition. A systematic review and meta-analysis. Aids.

[118] WHO (2020). Women Living with HIV Have a Six-Fold Increased Risk of Cervical Cancer When Compared to Women Without HIV.

[119] Wen X.J., Balluz L., Town M. (2012). Prevalence of HIV risk behaviors between binge drinkers and non-binge drinkers aged 18- to 64-years in US, 2008. J. Community Health.

[120] Chersich M.F., Luchters S.M., Malonza I.M., Mwarogo P., King’ola N., Temmerman M. (2007). Heavy episodic drinking among Kenyan female sex workers is associated with unsafe sex, sexual violence and sexually transmitted infections. Int. J. STD AIDS.

[121] Chersich M.F., Bosire W., King’ola N., Temmerman M., Luchters S. (2014). Effects of hazardous and harmful alcohol use on HIV incidence and sexual behaviour: A cohort study of Kenyan female sex workers. Glob. Health.

[122] Chiao C., Morisky D.E., Rosenberg R., Ksobiech K., Malow R. (2006). The relationship between HIV/Sexually Transmitted Infection risk and alcohol use during commercial sex episodes: Results from the study of female commercial sex workers in the Philippines. Subst. Use Misuse.

[123] Olusanya O.O., Wigfall L.T., Rossheim M.E., Tomar A., Barry A.E. (2020). Binge drinking, HIV/HPV co-infection risk, and HIV testing: Factors associated with HPV vaccination among young adults in the United States. Prev. Med..

[124] Oh H.Y., Kim M.K., Seo S., Lee D.O., Chung Y.K., Lim M.C., Kim J., Lee C.W., Park S. (2014). Alcohol consumption and persistent infection of high-risk human papillomavirus. Epidemiol. Infect..

[125] Oh H.Y., Seo S.-S., Kim M.K., Lee D.O., Chung Y.K., Lim M.C., Kim J.-Y., Lee C.W., Park S.-Y. (2015). Synergistic Effect of Viral Load and Alcohol Consumption on the Risk of Persistent High-Risk Human Papillomavirus Infection. PLoS ONE.

[126] Mekonnen A.G., Mittiku Y.M. (2023). Early-onset of sexual activity as a potential risk of cervical cancer in Africa: A review of literature. PLoS Glob. Public Health.

[127] Lytvynenko M., Bocharova T., Zhelezniakova N., Narbutova T., Gargin V. (2017). Cervical transformation in alcohol abuse patients. Georgian Med. News.

[128] Nielsen A., Munk C., Jørgensen H.O., Winther J.F., van den Brule A.J., Kjaer S.K. (2013). Multiple-type human papillomavirus infection in younger uncircumcised men. Int. J. STD AIDS.

[129] Ho G.Y., Bierman R., Beardsley L., Chang C.J., Burk R.D. (1998). Natural history of cervicovaginal papillomavirus infection in young women. N. Engl. J. Med..

[130] Burkett B.J., Peterson C.M., Birch L.M., Brennan C., Nuckols M.L., Ward B.E., Crum C.P. (1992). The relationship between contraceptives, sexual practices, and cervical human papillomavirus infection among a college population. J. Clin. Epidemiol..

[131] Sikström B., Hellberg D., Nilsson S., Mårdh P.A. (1995). Smoking, alcohol, sexual behaviour and drug use in women with cervical human papillomavirus infection. Arch. Gynecol. Obstet..

[132] Tolstrup J., Munk C., Thomsen B.L., Svare E., van den Brule A.J., Grønbaek M., Meijer C., Kjaer Krüger S. (2006). The role of smoking and alcohol intake in the development of high-grade squamous intraepithelial lesions among high-risk HPV-positive women. Acta Obstet. Gynecol. Scand..

[133] Huang J., Deng Y., Boakye D., Tin M.S., Lok V., Zhang L., Lucero-Prisno D.E., Xu W., Zheng Z.J., Elcarte E. (2022). Global distribution, risk factors, and recent trends for cervical cancer: A worldwide country-level analysis. Gynecol. Oncol..

[134] Chincha Lino O.J., Chinchihualpa Paredes N.O., Samalvides Cuba F. (2022). Factors associated with normal or abnormal Papanicolaou smear among HIV-infected women at a national hospital in Lima, Peru, 2012–2015. AIDS Care.

[135] Hayumbu V., Hangoma J., Hamooya B.M., Malumani M., Masenga S.K. (2021). Cervical cancer and precancerous cervical lesions detected using visual inspection with acetic acid at Livingstone Teaching Hospital. Pan Afr. Med. J..

[136] Abebe M., Eshetie S., Tessema B. (2021). Prevalence of sexually transmitted infections among cervical cancer suspected women at University of Gondar Comprehensive Specialized Hospital, North-west Ethiopia. BMC Infect. Dis..

[137] Mancuso P., Djuric O., Collini G., Serventi E., Massari M., Zerbini A., Giorgi Rossi P., Vicentini M. (2020). Risk of cancer in individuals with alcohol and drug use disorders: A registry-based study in Reggio Emilia, Italy. Eur. J. Cancer Prev..

[138] Allen N.E., Beral V., Casabonne D., Kan S.W., Reeves G.K., Brown A., Green J. (2009). Moderate alcohol intake and cancer incidence in women. J. Natl. Cancer Inst..

[139] Sigvardsson S., Hardell L., Przybeck T.R., Cloninger R. (1996). Increased cancer risk among Swedish female alcoholics. Epidemiology.

[140] Kjaerheim K., Andersen A. (1994). Cancer incidence among waitresses in Norway. Cancer Causes Control.

[141] Tønnesen H., Møller H., Andersen J.R., Jensen E., Juel K. (1994). Cancer morbidity in alcohol abusers. Br. J. Cancer.

[142] Adami H.O., McLaughlin J.K., Hsing A.W., Wolk A., Ekbom A., Holmberg L., Persson I. (1992). Alcoholism and cancer risk: A population-based cohort study. Cancer Causes Control.

[143] Alcohol Consumption and Cervical Cancer Associations Among Women in Los Angeles, California. https://scholarworks.waldenu.edu/cgi/viewcontent.cgi?article=6154&context=dissertations.

[144] Abdalla AE T.T., Gallagher J., Schmitt J.W. (2020). Alcohol Consumption and the Development of High-Grade Cervical Dysplasia. Gynecol. Obstet..

[145] Kingsley K., Struthers M., Freel N., Enyeart J., Munford W., Miller C.N., Spangelo B., Steffen J., Park G., Keiserman M.A. (2012). Ethanol and acetaldehyde mediate folic acid and human papillomavirus-induced proliferation of oral squamous cell carcinoma cells in vitro. BMC Proc..

[146] Arroyo Mühr L.S., Gini A., Yilmaz E., Hassan S.S., Lagheden C., Hultin E., Garcia Serrano A., Ure A.E., Andersson H., Merino R. (2024). Concomitant human papillomavirus (HPV) vaccination and screening for elimination of HPV and cervical cancer. Nat. Commun..

[147] El-Zein M., Richardson L., Franco E.L. (2016). Cervical cancer screening of HPV vaccinated populations: Cytology, molecular testing, both or none. J. Clin. Virol..

[148] Cervical Cancer. https://www.who.int/news-room/fact-sheets/detail/cervical-cancer.

[149] Luu X.Q., Jun J.K., Suh M., Oh J.K., Yu S.Y., Choi K.S. (2025). Cervical Cancer Screening, HPV Vaccination, and Cervical Cancer Elimination. JAMA Netw. Open.

